# A Qualitative Study to Explore the Barriers for Nonadherence to Referral to Hospital Births by Women with High-Risk Pregnancies in Nepal

**DOI:** 10.3390/ijerph18115801

**Published:** 2021-05-28

**Authors:** Sushma Rajbanshi, Mohd Noor Norhayati, Nik Hussain Nik Hazlina

**Affiliations:** 1 Women’s Health Development Unit, School of Medical Sciences, Universiti Sains Malaysia, Health Campus, Kubang Kerian 16150, Kelantan, Malaysia; sushmarajbanshi@yahoo.com (S.R.); hazlinakck@usm.my (N.H.N.H.); 2 Department of Family Medicine, School of Medical Sciences, Universiti Sains Malaysia, Health Campus, Kubang Kerian 16150, Kelantan, Malaysia

**Keywords:** nonadherence, barriers to referral hospital birth, homebirth, high-risk pregnancy, Nepal

## Abstract

Maternal and neonatal morbidity and mortality tend to decrease if referral advice during pregnancy is utilized appropriately. This study explores the reasons for nonadherence to referral advice among high-risk pregnant women. A qualitative study was conducted in Morang District, Nepal. A phenomenological inquiry was used. Fourteen participants were interviewed in-depth. High-risk women who did not comply with the referral to have a hospital birth were the study participants. Participants were chosen purposively until data saturation was achieved. The data were generated using thematic analysis. Preference of homebirth, women’s diminished autonomy and financial dependence, conditional factors, and sociocultural factors were the four major themes that hindered hospital births. Women used antenatal check-ups to reaffirm normalcy in their current pregnancies to practice homebirth. For newly-wed young women, information barriers such as not knowing where to seek healthcare existed. The poorest segments and marginalized women did not adhere to referral hospital birth advice even when present with high-risk factors in pregnancy. Multiple factors, including socioeconomic and sociocultural factors, affect women’s decision to give birth in the referral hospital. Targeted interventions for underprivileged communities and policies to increase facility-based birth rates are recommended.

## 1. Introduction

The World Health Organization has defined the concept of compliance as the accomplishment of certain behaviors, such as taking prescribed medication, following a diet, executing lifestyle changes, and following the recommendations provided by healthcare providers [1]. Thaddeus and Maine’s three delays model is the foundational model for studying delay in compliance [2]. According to this model, nonadherence to a referral for facility-based birth can be considered the first delay in decision making to seek care [2]. The referral process represents the handing over of care from a general practitioner to a specialist [3]. Referral during pregnancy is essential to ensure that women with high-risk pregnancies and complications access immediate and appropriate care [4]. The adherence process requires both the patient and the healthcare providers’ involvement and good communication among all involved parties. 

Noncompliance with medical treatment is not unique to women with high-risk pregnancies [5]. Pregnant women with risk factors did not comply with the referral advice with the same seriousness as a referral in the event of a complication during childbirth [6]. Inconsistencies between risk appraisals made by pregnant women and healthcare providers have been noted as reasons for nonadherence [7]. Pregnant women and their relatives may not accept a referral when they have seen other women with the same problem giving birth safely at home after being referred [6]. 

The magnitude of high-risk pregnancies in Nepal was 41.0% in 2000 [8], 24.6% in 2015 [9], and 14.4% in 2020 [10]. The maternal mortality ratio is 239 per 100,000 live births, the perinatal mortality rate was 31 per 1000 live births, and the neonatal mortality rate was 21 per 1000 live births in 2016 [11]. Based on a national protocol for pregnant women, the achievement for completed four antenatal care (ANC) visits was 56% [12]. The institutional birth rate was 63.2%, of which 18% of births were conducted by cesarean section, which was as high as 30% in Province 1 [12]. Women from poor and deprived communities are not utilizing the services that they should [13].

Maternal and newborn morbidity and mortality are expected to decrease if referral during pregnancy is utilized appropriately [14]. Many factors are involved in patients’ noncompliance with facility-based birth, which is a major problem that prevents healthcare providers from achieving the desired outcomes of increasing the facility-based birth. From the women’s perspective, their perceptions of the quality of care at health facilities could influence their adherence to a referral [6].

The success of ANC programmes on risk screening is the utilization of referral hospitals by high-risk women [15]. Contradictory to this expectation, the literature showed that about 32% to 43% of women referred during routine ANC for a hospital birth did not adhere to the referral advice [8,16]. Women’s nonadherence to referrals to the tertiary hospital childbirth due to their high-risk status has received scant attention. The purpose of the present study was to explore the barriers to nonadherence to referral hospitals in pregnant women with high-risk pregnancies [16].

## 2. Materials and Methods

### 2.1. Study Design

A qualitative phenomenological research inquiry was used. Morang District, which is in the eastern region of Nepal, was purposively selected for its high population density, diverse ethnicity, and urban and rural mixed population balance. Data were collected from November 2019 until April 2020. A topic guide was designed based on the study objectives. Giving birth at the birthing center or homebirth instead of referral hospital after referral advice from the primary care level was regarded as nonadherence in this study. Fourteen mothers with high-risk factors who gave birth at home or the birthing center were interviewed in-depth. 

### 2.2. Study Settings

The Morang District is the second most highly populated district of Nepal. The total population of this district is 1,073,307, and the total expected pregnancies 27,799 in 2020 [12]. A mixture of both public and private health services is used in Nepal. Maternal health care services, that is, ANC and birthing services, are provided free of charge. The public health services are provided through three hospitals, 23 birthing centers, and 44 health posts in the Morang District [17]. The facility-based birth rate in Province 1 where Morang district is situated is 62% [12]. Comprehensive emergency obstetric and newborn care is provided by the hospitals and basic emergency obstetric and newborn care by the birthing centers via skilled birth attendants [18]. Birthing centers operate 24 h a day and have one bed. Normal, uncomplicated pregnancies or low-risk pregnancies can be handled at birthing centers [18]. 

The birthing centre provides seven basic services, which include administration of parenteral antibiotics, uterotonic drugs, parenteral anticonvulsants, manual removal of the placenta, removal of retained products, assisted vaginal birth, basic neonatal resuscitation [19]. The birthing center used in this study refers to a community-level health institution that provides basic emergency obstetric and neonatal care. The referral hospital refers to hospitals with comprehensive emergency obstetric and neonatal care that provides cesarean section, blood transfusion, and neonatal resuscitation in addition to the seven basic services [19]. Women requiring other than basic emergency obstetric and neonatal services are referred to the referral hospital with comprehensive emergency obstetric and neonatal services.

### 2.3. Sample and Recruitment Procedures

Postpartum women who had high-risk factors during their pregnancy—based on the Malaysian risk stratification approach [20]—postpartum women within 42 days of birth who had given birth at home or the birthing center with basic emergency obstetric care and nonadherence to referral hospital birthing advice, were eligible to participate. All 23 birthing centers in the Morang District were visited to find study participants. A purposive sampling method was used. Healthcare providers from the birthing centers were the first contact point, and female community health volunteers were the second contact point to enquire about eligible participants. The participants were located in closed community settings where minority ethnic groups like Santhal or Muslim or Dalits reside. The interviews were conducted at the participants’ homes or at the birthing center, whichever the participants felt more comfortable with. 

### 2.4. Data Generation

Women were approached for in-depth interviews and written informed consent at their homes. Verbal approval was taken before using a tape recorder. Field notes were taken during the interviews when necessary. Written informed consent of the guardian was received for minor participants. The interview was conducted by the principal investigator (PI) using a topic guide with open-ended questions. Probing or follow-up questions were asked when necessary. The interviews were conducted in the Nepali language; for two participants who cannot reply in Nepali, a female community health volunteer was used as a translator. The interview duration was 25–40 min. The interviews continued until the information provided by the participants reached a saturation point [21].

### 2.5. Data Analysis

With the field notes, verbatim transcriptions of tape-recorded interviews were transcribed directly into the English language to ease coding and analysis. The participants’ identities were anonymized during the transcription phase. QSR International Pty Ltd (2018) NVivo (Version 12) was used to systematically review and code the data, and thematic data analysis was performed. English translated transcripts were prepared for analysis. The transcripts were familiarized by rereading. Every line or paragraph of the transcripts was inductively systematically coded based on the emerging codes that fit with the concept suggested by the data [21]. To ensure credibility, English transcripts were shared among the authors to perform independent coding and then discussed for their consistency of views. Initial codes were discussed for similar understandings and interpretations. Deductively, codes were then grouped into categories of subthemes and finally were manually grouped into emerging themes. The themes were revisited when reviewing the transcripts to find additional supporting evidence and to understand the contexts of the codes [21]. Literature reviews helped further clarify similar themes. Themes were renamed and discarded several times to come up with suitable themes. The codes were grouped into suitable subthemes and themes, which were revisited several times until the data provided a meaningful interpretation. Finally, the codes were reread and summarized in narratives, and appropriate quotes from the participants were added. 

### 2.6. Trustworthiness

For the credibility of this study, the PI was involved in interviewing the women and transcribing the data. During data collection, the PI could understand the participants’ contexts through direct field observations, interviews, and informal inquiries with the healthcare providers from the 23 birthing centers and female community health volunteers, which increased the study’s credibility [22]. The participants were recruited from multiple sites of the district, which also adds to the trustworthiness of the triangulation of the data [22]. Three authors were involved in the data analysis process. The coded transcripts in NVivo were shared with all co-authors, who reviewed them critically and discussed if self-bias was introduced; the authors then referred back to the transcripts and codes for verification. Direct quotes and narrations of the participants were reported. 

## 3. Results

### 3.1. Characteristics of Participants

An in-depth interview was conducted with 14 postpartum women who were identified as having experienced high-risk pregnancies. Nine out of 14 births (64.3%) were homebirths, and five were born at birthing centers (see Table 1). Pregnancies or births of high risk faced by the participants were prolonged fever in pregnancy, prolonged labor pain of four days, preterm birth, retained placenta, multigravida (seventh child), postdate exceeded by >7 days, growth of tumor together with the fetus, and loss of newborns. Two participants had lost their newborns. These women, mostly from the Santhal and Muslim communities, lived close and one community setting. The majority of participants (10 of 14) in our study represent women from minority ethnicities and/or underprivileged groups. They shared common norms of practicing homebirths. Details of the participants’ characteristics are listed in Table 1. 

### 3.2. Barriers for Nonadherence

Four themes emerged from the thematic analysis regarding the reasons for nonadherence and homebirth or birthing center-only birth among high-risk pregnant women: (i) preference for homebirth, (ii) lack of women’s autonomy and financial dependence, (iii) conditional factors and (iv) sociocultural factors. 

#### 3.2.1. Preference for Homebirth

##### The Women’s Intention was to Give Birth at Home

Most of the participants had witnessed their relatives or neighbors having given normal homebirth, finding childbirth normal and natural. Eight of the participants’ intention was to give birth at home, and only a few participants intended hospital or birthing center births. Home comfortable environment and family support were participants main reason to give homebirth. Not to mention, women could look after household chores if they were at home.

The participants were thankful to have given birth at home normally without having to leave their homes. A few women were grateful for homebirth as that they did not have to be operated. Two of the participants who were rushed to the birthing center, wished to give homebirth.

*My wish was to give birth at home, it’s better at home, if you go to the hospital, you have to endure some pain and if one givesing birth at home. I was happy if it [childbirth] was at home.*

(P0014)

There was a common understanding that women should first wait and try normal childbirth at home; otherwise, the second option was to rush the woman to the nearby birthing center. Participants expressed that they believe in the institutional capacity to handle an emergency.

*Where to give birth? If you can give birth at home, then give birth at home, if you cannot, and then go to the hospital.*

(P003)

##### Trust in Healthcare Providers’ Word, Ultrasound and Medical Test Reports

Healthcare providers whom the women trusted were local ones; they played a key role in pregnant women’s health-seeking practice. Healthcare providers were among other sources for informants. Pregnant women listened and tried to follow healthcare providers’ advice, given mostly during ANC.

Even though the women practiced homebirth, “home call” was also practiced. Trusted local healthcare providers who were by profession, either auxiliary healthcare providers or midwives, were called to assist in homebirths, especially in the Muslim community. Understanding the local custom and practice, in these community settings where homebirth is practiced, few healthcare providers were found to assure and encourage homebirth.

Pregnant women had good knowledge about the use of ultrasound and got ultrasonography from private clinics, even if they had not completed their required four ANC visits. The healthcare providers’ assurance of “everything was fine with the baby” after ultrasonography and medical test results was a huge relief to practice home birth. It was one of the reasons that further encouraged homebirth practice. Reasons for homebirth given by one of the participants:

*In the ultrasound report, it stated that everything is fine, the baby is fine, and I was told that it would be fine …*

(P003)

##### Family Members were Decision-Makers; Pregnant Women Had a Passive Role in Decision Making

Mothers and mothers-in-law—sometimes even aunts—made the decisions about the place of birth, especially in the case of the young participants. Most of the participants’ husbands were working away from home or worked abroad. Hence, family members were the ones who would support and take care of the women in their pregnancy and birth. The husband’s role in birth decision-making was noninfluential, and few participants even mentioned they were unsupportive. The younger participants usually played passive roles and let their family members make decisions for them. For example, two participants were not persuasive enough and agreed with their family members’ decision to give birth at the nearest birthing center, although they were referred for hospital birth.

*If she cannot give birth, she will be taken outside [birthing center], but there was only slight pain, so she stayed at home … Kept her [participant] at home during the night, the next day, when it was 10 am, the pain got intense. Then we called the ambulance took her to the birthing center.*

(P004 participant mother)

It was a general practice within the participant’s family that neither discussions about the place of birth nor birth preparation were held. Arranging transportation in advance or keeping the ambulance service’s contact number in case of emergency were also not practiced. Trusted relatives recommended referral centers for the place of birth if the complications arose. One of the participants lost her baby because of a family members’ late decision to transfer the pregnant woman to another institution. 

One of the significant reasons for nonadherence to the referral hospital was that the family members would decide for the participants for the place of birth. The convenience of having the birthing center nearby was sometimes a hindrance to the women’s adherence to the referral hospital. 

#### 3.2.2. Lack of Women’s Autonomy and Financial Dependence

##### Fear of Hospital Environment, Past Negative Experiences, Privacy Issues, and Rumors

Nonadherence to hospital birth was because the participants were scared of the hospital environment. They were scared of taking injections. For some, even thinking about the hospital made their hearts pound faster. Hence, participants were terrified that they might be operated on if they went to the hospital. This may have been intensified by the rumors that caesarean sections are mostly done if one’s labor pain does not progress in hospitals. 

Participants’ previous birth experiences had led to both positive and negative behavior changes found in health care services utilization. One participant had a stillbirth in her previous pregnancy at the hospital. This experience changed the participant’s and family members’ perception that the outcome could have been different, and they decided to have their current baby at home. After homebirth, another participant lost her newborn within a few days and decided to give birth at the health institution.

Three participants specifically mentioned that they preferred homebirth because they felt shy of male staff in the hospital, which hindered these women from visiting health facilities. Another reason mentioned was women had heard about the rumor that “many nurses will insert their hands inside the pregnant woman’s body in the hospital.” For few participants, it was an issue of their dignity. They asked why they should showcase their private parts in front of everyone. One of the participants preferred giving birth alone, without anyone’s presence, not even their women relatives.

*At the hospital, [laughs] I heard that they would tie the legs and hands [laughter]… And when one has normal birth, then also, they will cut a bit. Since then, I am afraid [laughter].*

(P0014)

Rumors of the hospital staff’s rude attitude and behavior were widespread. One participant did not make any noise during labor pain in her previous hospital birth because she had heard rumors that the nurses usually beat the women who made noise. Rumors about being unnecessarily operated on at the hospital were also widely believed. 

##### Unfamiliar Place and Lack of Knowledge of the New Environment

Eight of the interviewed participants were newly married, younger-aged women. Three of the participants were unmarried but were living at their partner’s home after disclosing their pregnancy. Hence, participants mentioned they found the husband’s residence new and unfamiliar. In the village context, they had not explored the area, and they did not know where to seek healthcare. They mentioned spending most of their time doing household chores and not having time to visit relatives’ houses. Even within the household, some participants mentioned they had not opened up with other family members.

Three participants found out that their community’s health facility was about 10 to 15 min away from where they lived or that the outreach clinic was available near their home only during the interview. These participants were getting ANC services from the next village, which was about 30 min walking distance. 

Women who gave birth at the birthing centers did not know that they would receive cash incentives and a set of clothes for the mother and newborn. They were surprised and joyful when they received it. 

##### Lack of Transportation, Poverty and Distance

Infrequent access to public transportation was a problem where the participants resided. The public bus would pass through the village a couple of times a day, a cheaper form of transportation, directly connecting the villages to the larger cities; otherwise, other options were electric vehicles, motorbikes, and bicycles. However, during an emergency, public transportation was useless.

*Here, to give birth at the birthing center, there are no transportations. So, people here give birth at home because of the scarcity of transportation services.*

(P005)

The women faced difficulty getting transportation if the labor pain started late at night. Few women lived in a deprived community, so they mentioned that they did not even own a bicycle. The participants said that the transportation charges during emergencies at night were higher than the daytime rates. Ambulances were available where the women lived, either from the public health facility or hiring privately. Although ambulance services were present, the participants mentioned that their charges were high and unaffordable.

*There is an ambulance service, but if we sometimes summon an ambulance. They charge Rs. 1500 (≈14.3 USD).*

(P005)

#### 3.2.3. Conditional Factors

##### Labor Pain Started at Night, Sudden Onset, and Short Labor 

Women who gave birth at home frequently mentioned a sudden onset of labor that started late at night. Because it was winter and cold at night and unavailability of transportation, family members, including their husbands, decided to wait until morning. Then, early in the morning, with intense pain and short labor, the participants mentioned that the baby was already born.

*Just like while working [household chores], for a while, … there was slight pain at 1 am, I felt that I wouldn’t give birth tonight … as the night progressed then the pain intensified, … then at 9:10 am, the baby was born.*

(P0014)

For a few participants, although they were referred to the referral hospital, they were taken to the nearest birthing centers by family members for two reasons: first, it was late at night, and second, the women had not communicated to their family members about their referral advice given by the healthcare providers. Reasons provided by one participant was:

*I don’t know [laughs]. There won’t be anyone at home. I am usually alone, that’s why… Yes, family members are there, but I usually don’t talk with anyone.*

(P0013)

##### Labor Pain Ahead of the Expected Date of Birth

Birth ahead of the expected dates of birth and first-time mother’s inexperience of recognizing labor pain was a hindrance to having a facility-based birth. Young inexperienced participants did not know if their pain ahead of the date of birth was labor pain or not. Their family members, especially their mother or mother-in-law, had to confirm their labor pain. Younger women mentioned that they endured the pain and did not mention it to their husband a few weeks or sometimes a month remained before the date of birth. Still, because of the confusion with the expected date of birth, the participants mentioned giving birth at home, a few were taken to the birthing center.

*It was not yet the time of birth that time. The doctor gave few additional more days, and I forgot the exact menstrual period missing date.*

(P002)

#### 3.2.4. Sociocultural Practices

##### Births Attended by the Traditional Birth Attendants and Consulting Spiritual Healers

Traditional birth attendants (TBAs) were called “grandmothers” in the village. The husband or family members summoned them once labor pain started. They were available anytime and did not charge any money. These TBAs were sometimes the women’s close relatives, such as a mother-in-law or aunt. If the TBAs were not confident and if the woman’s situation started getting worse, they would ask the family members to take the pregnant woman to a health institution.

*At night, now nothing, mother-in-law heated some hot water and gave it to me to drink. The grandmother [traditional birth attendant] was there who used to come if there is labor pain at home for support in childbirth … but I could not give birth, that’s why I was taken there [birthing center].*

(P0012)

Spiritual healers called ”Dhami” were consulted for pregnancy-related problems, infertility, and even before taking medicines. Although spiritual healers and TBAs had major roles in women’s pregnancy and childbirth, they did not directly influence birth place decisions. The women believed in witchcraft and being under bad omens. Spiritual healers were the first place that the women consulted; sometimes, these healers were the last option for women if unsatisfied with healthcare providers’ consultation. 

##### Role of Women Relatives

The woman’s mother, mother-in-law, sisters-in-law, friends, aunties, and other women relatives had a bigger role during the participant’s place of birth. The mothers and mothers-in-law were the ones who recognized the participants’ signs of labor pain after hearing about the participants’ conditions. For women who did not have a mother-in-law, aunties or sisters-in-law took their role and provided suggestions, sometimes taking them to the health institution. There was a culture among the Santhal community that a sister-in-law would cut the umbilical cord upon the birth of the baby. For this reason, one of the participants was taken to her in-law’s house support childbirth and fulfill this tradition.

*I was scared, and afterward, my mother-in-law told me that it is labor pain. The aunties here also asked what is happening, and I told them I am having this [explain her pain]; then they [aunties] told me it means you are having labor pain.*

(P0012)

The participants consulted about their pregnancy problems and sought advice from them; the participants tended to listen to a relative’s advice, and they were their source of assurance. Some of the participants were likely to choose the same health facility suggested by their relatives because it was recommended based on their personal experiences.

## 4. Discussion

Our study found that women displaying nonadherence to a referral hospital mostly belonged to underprivileged and marginalized groups. The decision behind nonadherence to referral for a hospital birth was a complex process, where economic, cultural, societal, and individual factors and the interactions between them played a key role. In a few other studies, women who were noncompliant to referral services were those from minority groups, such as ultra-poor communities, immigrants, and socially disadvantaged groups [23,24]. There is increasing evidence that women of marginalized population groups, such as those living in areas of deprivation, women from ethnic minority groups, refugees, substance abusers, and those from traveling communities, do not use health services, even though these services are accessible and affordable to them [25]. Similar to our study, in India, women from the lower socioeconomic strata with less education were disproportionately more likely to give birth at home [26]. Similar to our study, younger women tend to give birth at home [27].

Participants found the home environment safe and comfortable and intended to give birth at home in family members’ presence in our study. Homebirth also addressed the privacy issue of a few participants. In Ethiopia, the women preferred homebirth because they believed facility-based birth was unnecessary and customary [28]. Some highly educated women perceive that homebirth is a safer option than hospital birth [29,30]. In some high-income countries, free birth and high-risk homebirth are becoming popular [30]. In the USA, women who had a planned homebirth had high satisfaction related to the home is a more comfortable environment [31,32]. For most women, home is a peaceful and restful place where they have more control over the events and environment [31]. 

However, when high-risk women from high-income countries chose homebirth, they had a “holistic” community midwife to support their homebirth [30]. One reason for the women from high-income countries choosing homebirth was general dissatisfaction with the birthing options offered within the mainstream maternity care system [33]. For some women in low-income countries, it was shown there was trust in the medical ability of the TBAs, and the women believed they would get the necessary support from family members if they chose homebirth [28]. Our study participants were not empowered or highly educated, nor were they aware of their health rights like the women from high-income countries. They preferred the home comfort to give birth; they had witnessed their relatives and neighboring women giving birth naturally. 

Women from the same ethnic groups were practicing homebirth in the same locality in our study. Gage et al. have pointed out the contagion process of exchanging and imitating behavior, information, and advice between social networks of better and less educated residents regarding appropriate care during pregnancy and childbirth [34]. Similarly, the study further added that communities with a high concentration of poor households and a low concentration of well-educated residents are likely to share intergenerational norms and cultures surrounding pregnancy care and practice [34].

The women’s perception was “if there is no complication, then one can give birth at home; if anything happens, one can always be rushed to the hospital”. The participants expressed similar sentiments in Kenyan and Bolivian studies [35,36]. The community perception was that facility-based birth is a service only utilized when complications arise [35,36]. In Uganda, the women had a belief that abnormal pregnancies should be born in the health facility [37]. 

The women did value the importance of ANC in our study, unlike the findings from other low-income countries, where the women did not feel the need to seek professional care when there was nothing wrong with their pregnancy [38]. Most of the participants adhered to all the medical investigations, routine check-ups, and ultrasonography. Once assured of a normal pregnancy, the women who culturally practiced homebirth stayed at home, supporting the findings from other studies that women preferred homebirth if assured of normal pregnancy [14,35,39]. In Tanzania and Uganda, supporting our study’s findings, mothers go to ANC to confirm a normal pregnancy to feel free to give birth at home [37,40]. 

Women were often dependent on family elders and husbands’ consent to comply with referral advice and depended on their financial support [34,39]. Studies from Nepal and Tanzania support this study’s evidence that mothers-in-law had a strong influence on their daughters-in-law’s uptake health services, were decision-makers, and women had little influence in maternal referrals [41,42]. Another study from Nepal also found that healthcare expenses were controlled by family members [43]. The woman usually has no active role but waits for others’ decisions [6,28]. 

Evidence from several studies has found that among women who did not comply with referral advice, financial constraints were a major reason mentioned [23,24,39,40,44,45,46], which is similar to our study. None of our participants was earning money, which is identical to another study in Nepal [41], thus putting these women in a lower status within the household hierarchy when making decisions for themselves. 

Where patriarchal systems were dominant, women have been found to be financially dependent on their husband or husband’s family [25]. The mother-in-law holds strong decision-making powers regarding pregnancy and birth because men have little knowledge and think it is a woman’s issue [46,47]. The participants were young, newly married, and inexperienced in pregnancy, which must have influenced the homebirth decisions by family members in our study. A mother’s age and education at childbirth have been shown to be important independent factors in determining the place of birth [42,48]. 

Younger women had less household decision-making power than older women [49]. Marital status can also influence birthing place, including for recently married women [14], similar in our study. This could have influenced the women’s autonomy and influenced their giving birth at home. Living in an extended family may also have influenced the decision-making power of the women [14]. The majority of the participants in our study had taken a passive role in decision-making for a place of birth. Unlike our study’s findings, Harrison et al. found that most women with a high-risk pregnancy wanted to actively participate in health decision-making, but not all preferred active participation [50]. 

One of the significant constraints faced by mothers in seeking health services was a lack of transportation [23,45,51,52] and lack of money for this [28,42,45,51]. A lack of or unaffordable transportation were barriers for nonadherence; similar to the findings in Kenya [35], India [53], and Bolivia [36]. Besides cash for transportation, hospital expenses and lodging expenses were also hindrances for facility-based deliveries [28]. At times, the perception that facility-based births could be expensive acted as a barrier to seeking services [26,38,39]. 

One of the main reasons mentioned for homebirth was labor pains started in the night and gave birth within a few hours in our study. Similar reasons were mentioned in other studies: labor started suddenly, and the child was born [37,42,44]; sudden labor occurred at night or unexpectedly [37], and there was no time to go to the health facility [37]. 

In Zambia, for women who visited ANC, only 45% knew their expected date of birth. During focus group discussions, the women said that the healthcare providers usually do not give the expected date of birth. Limited awareness of the date of some mothers’ last menstrual period can also play a role [54]. In our study, a few participants gave birth at home because their labor pain started preterm, and because the participants were young, they waited to say this and gave birth at home.

Women living in rural areas who mainly were busy with household chores, and farming found the hospital environment to be very unfamiliar and fearful. Similar conditions were found in Tanzania and India, where rural women feared the unknown urban environment and unfamiliar surroundings [39,53]. Several other studies have shown that women find hospital stays frightening because of the hospital staff, surroundings, medications, and routines [39,55]. 

Our participants mentioned fear of cesarean as a reason for avoiding facility-based birth. They said that they even were scared of taking vaccines, and the thought of being operated on scared them. This finding was similar to other studies [35,39]. There was an unfounded rumor in Ethiopia that every woman gets operated on in a health facility [28]. Rumors can harm institutions [56], and rumors of bad incidents spread quickly in communities [35]. Secondhand knowledge can affect women’s decision-making in the future [35]. In our study, rumors of staff beating and tying the women to bed threatened already scared women to have facility-based births. 

The women felt embarrassed and shy about male healthcare providers in the hospital, which was one reason for nonadherence to hospital birth. Like this finding, in Tanzania and India, some young women did not have facility-based births because of male healthcare providers’ presence [34,42,53]. In contrast, a qualitative study in Ethiopia found that the participants preferred male healthcare providers over female healthcare providers [56]. The reasons for their preference were because male providers were caring, polite, respectful, soft-hearted, and fair in every respect [56].

TBAs or friends/relatives were the most common persons during home birth [24,35]. These TBAs were readily available anytime and lived nearby; they were consulted for pregnancy-related problems, supported by other studies [25,35]. Interestingly, they do not charge money; their payment was negotiable, which could be paid in cash or kind or on credit, similar to a study in Kenya [35] and Uganda [37]. In Ethiopia, the women believed that the TBAs could handle normal births and attempt home birth [28]; otherwise, the women can go for facility-based births. The women turned toward TBAs because they were cheaper than the perceived high cost of facility-based deliveries [35]. 

In our study, some communities called healthcare providers at home to assist in homebirth. Some of these healthcare providers encouraged homebirth understanding local customs. In Bolivia, the practice of calling for a paid midwife [36], similar to our study, existed. In India and Bangladesh, purchasing pain-relievers, labor-inducing medicines, and receiving saline with an injection (oxytocin) from a pharmacy and private doctors were practiced [24,53]. 

Husbands were the major financial supporters; thus, they were involved in decision-making about the place of birth [57]. The husbands were not the final decision-makers if they were living in a joint family. The passive role or unsupportive role of a husband was a barrier to service access in our study and some other studies [24,58]. 

For the women, the decisions regarding the place of birth are strongly influenced by their relatives’ advice [59]. Many women turned to their mothers, mothers-in-law, aunts, and sisters for support and advice during pregnancy, birth, and after birth [60]. The women listened to their family members’ and relatives’ advice of when and where to obtain health services [60], which was also found in our study.

Women did not adhere to biomedical risk factors in a mismatch between biomedical risk factors and community perceived risk [39]. It was usually found that the community does not often agree with referral advice of expectant mothers below 20 years and multigravida with five or more children if they had no previous complication [6,47]. In our study, nulliparity and younger age were not considered high risk. 

## 5. Strengths and Limitations

The data were collected firsthand, ensuring no loss of participants’ data and increasing the accuracy of interpretations without losing the essence of the content during the translation from audiotape to transcription. The women were purposively sampled after visiting 23 birthing centers in the Morang District, representing the minority communities. 

The interview data lack triangulation by other qualitative data collection methods to validate the women’s accounts. Indeed, the women’s perceptions and personal reasons for barriers could not be validated by an external person. Variations in age, education, and socioeconomic status could not be sought due to challenges in finding eligible participants. Data collection was based on a one-time interview with the participants who happened to be underprivileged groups. They were also young, laughed a lot or were very quiet, needing assisted exploration with participants’ guardians such as mothers. Some participants communicate in the local dialect; thus, female community health volunteers were used as translators. 

## 6. Conclusions 

Women from underprivileged and minority groups were found to be practicing homebirths. The decision of nonadherence to a referral to a hospital among high-risk pregnant women was complicated, and multiple factors played their role. There were multiple issues with hospitals as reasons for nonadherence, as fearing the hospital environment, past negative experiences, and rumors. Our participants preferred homebirths and assured normalcy in their pregnancy to practice homebirths. Few healthcare providers practicing home calls to assist births were found encouraging homebirths.

## 7. Recommendations

Targeted interventions for a closed community setting of ethnic minority groups, such as the Dalit, Santhal, and Muslim communities, should be introduced to increase facility-based births. Healthcare providers should prioritize and encourage facility-based birth to younger pregnant women and ask if they have any inquiries about their pregnancy. A lack of transportation and financial constraint was frequently mentioned; therefore, subsidized ambulance services should be an option, as the participants have suggested. The right to information and removing informational barriers should be addressed among women living in marginalized communities. 

## Figures and Tables

**Table 1:** Background characteristics of the participants (*n* = 14).

Characteristics	*n*	(%)
Ethnicity Muslim Santhal Terai Madhesi Janajati Dalit	5 4 2 2 1	(35.8) (28.5) (14.3) (14.3) (7.1)
Age (years) ≤18 19–24 25–29 30–34	8 4 1 1	(57.1) (28.6) (7.1) (7.1)
Marital status Married Unmarried	11 3	(78.6) (21.4)
Type of family Joint Nuclear Extended	10 3 1	(71.4) (21.4) (7.1)
Education None Primary Secondary Higher secondary	3 7 3 1	(21.4) (50.0) (21.4) (7.1)
Occupation Housewife	14	(100)
Husband occupation Labour work Sales Agriculture	9 3 2	(64.3) (21.4) (14.3)
ANC * visits <4 ≥4	8 6	(57.1) (42.9)
Parity Primipara Multipara	8 6	(57.1) (42.9)
Place of birth Homebirth Birthing centre	9 5	(64.3) (35.7)

Note: * antenatal care.

## Data Availability

The authors are happy to share anonymized data related to this paper upon receiving a special request, along with the purpose of that request. Interested parties may contact hayatikk@usm.my.
